# Single-cell transcriptome sequencing provides insight into multiple chemotherapy resistance in a patient with refractory DLBCL: a case report

**DOI:** 10.3389/fimmu.2024.1303310

**Published:** 2024-03-12

**Authors:** Kewei Zhao, Qiuhui Li, Pengye Li, Tao Liu, Xinxiu Liu, Fang Zhu, Liling Zhang

**Affiliations:** Cancer Center, Union Hospital, Tongji Medical College, Huazhong University of Science and Technology, Wuhan, China

**Keywords:** diffuse large B-cell lymphoma, single-cell RNA sequencing, treatment resistance, tumor heterogeneity, tumor immune microenvironment

## Abstract

Relapsed and refractory diffuse large B-cell lymphoma (DLBCL) is associated with poor prognosis. As such, a comprehensive analysis of intratumoral components, intratumoral heterogeneity, and the immune microenvironment is essential to elucidate the mechanisms driving the progression of DLBCL and to develop new therapeutics. Here, we used single-cell transcriptome sequencing and conventional bulk next-generation sequencing (NGS) to understand the composite tumor landscape of a single patient who had experienced multiple tumor recurrences following several chemotherapy treatments. NGS revealed several key somatic mutations that are known to contribute to drug resistance. Based on gene expression profiles at the single-cell level, we identified four clusters of malignant B cells with distinct transcriptional signatures, showing high intra-tumoral heterogeneity. Among them, heterogeneity was reflected in activating several key pathways, human leukocyte antigen (HLA)-related molecules’ expression, and key oncogenes, which may lead to multi-drug resistance. In addition, FOXP3+ regulatory CD4+ T cells and exhausted cytotoxic CD8+ T cells were identified, accounted for a significant proportion, and showed highly immunosuppressive properties. Finally, cell communication analysis indicated complex interactions between malignant B cells and T cells. In conclusion, this case report demonstrates the value of single-cell RNA sequencing for visualizing the tumor microenvironment and identifying potential therapeutic targets in a patient with treatment-refractory DLBCL. The combination of NGS and single-cell RNA sequencing may facilitate clinical decision-making and drug selection in challenging DLBCL cases.

## 1 Introduction

Diffuse large B-cell lymphoma (DLBCL) is a highly heterogeneous malignant tumor with regard to clinical features, histological morphology, and genetic and molecular phenotype. Although standard first-line treatment with R-CHOP can cure DLBCL in 60% of patients, 40% of patients remain refractory to treatment or relapse after remission (1). The exact mechanisms driving disease relapse or refractoriness remain largely unknown, which can present a barrier to selecting appropriate treatment options.

Our understanding of the pathogenesis and progression of lymphoma has expanded considerably with knowledge of genetic alterations and dysregulation of intracellular pathways (2). By contrast, the role of the microenvironment in B-cell lymphoma has been underestimated. It is important to note that since B cells are an important part of the normal functioning immune system, the interactions between malignant cells and immune cells in the tumor microenvironment of B-cell lymphomas are more complex than in other solid tumors (3). The lymphoma microenvironment (LME) is a complicated interaction network of tumor, immune, and stromal cells with intra- and inter-tumor heterogeneity. Moreover, cytokines and chemokines secreted throughout the LME transmit various tumor-promoting and tumor-suppressing signals to regulate tumor growth and evolution, thereby affecting tumor progression and response to immunotherapy (3, 4). As a result, the LME is increasingly becoming a focus of attention in B-cell lymphoma pathophysiology and treatment resistance research.

Single-cell RNA sequencing enables comprehensive characterization of the cellular compositions and transcriptional features of malignant cells and infiltrating immune cells in many types of cancer (5). In order to explore the heterogeneity of DLBCL, decode the components of DLBCL tumor microenvironment and intratumor crosstalk of distinct cells, we conducted single-cell transcriptomic analysis of a patient who was resistant to multi-course chemotherapy, hoping to find some evidence related to tumor malignant progression and drug resistance.

## 2 Case presentation

A 71-year-old female patient presented with a painless and growing right supraclavicular mass in December 2020. The patient underwent ultrasound-guided puncture biopsy at Union Hospital, Tongji Medical College, Huazhong University of Science and Technology on February 1, 2021, and postoperative pathology showed the following: CD5-positive diffuse large B-cell lymphoma of germinal center B cell (GCB) origin with dual immune-expression of BCL2 and C-MYC. Immunohistochemical staining identified the following proteins in tumor cells: CD20 (+), CD3 (-), CD19 (+), CD22 (+), CD5 (+), CD10 (+), BCL6 (+), MUM1(+), BCL2 (+), C-MYC (60+), P53 (60%+), Ki67 (LI:90%) and EBER ISH (-). Fluorescence *in situ* hybridization (FISH) showed a negative C-MYC/IgH gene fusion test. Further PET/CT imaging was performed that showed the following: huge soft tissue masses in the right superior/inferior clavicle region, chest wall muscle space, right armpit, involving the right pectoralis major and pectoralis minor, multiple enlarged lymph nodes in the right neck, right armpit, left thoracic wall muscle space, left side of the erectus spinalis, mediastinum 2R region, right side of the sternum, and left medial psoas major, with partial fusion and abnormal increased metabolism. The spleen was enlarged and localized metabolism was increased. We also identified a soft tissue mass in the left iliac socket, involving the left iliopsoas muscle, piriformis muscle, and obturator internus muscle, with an abnormal increase in metabolism. These lesions were considered malignant lymphoma infiltration. There were no obvious abnormalities in bone marrow cytology and immunotyping. The patient was eventually diagnosed with CD5-positive DLBCL, GCB, stage IV, IPI score 4, with BCL2 and C-MYC dual expression.

Subsequently, the patient received four cycles of R-CHOP starting in February 2021, and a partial response (PR) was evaluated after two cycles of chemotherapy. After four cycles of chemotherapy, the patient developed a new mass in the right chest wall, and PET-CT indicated a progression of disease (PD). On June 23, 2021, the patient received combined rituximab, lenalidomide and zanubrutinib for 1 cycle. However, the mass in the patient’s chest wall continued to increase in size. On August 4, 2021, the patient underwent a second ultrasound-guided puncture biopsy of a recurrent right chest wall mass. The specimens were sequenced by bulk next-generation sequencing and single-cell transcriptome sequencing. The patient was then enrolled in the ATG 010 clinical trial in August, 2021 (protocol No: Atg-010-DLBCL -001). Unfortunately, after 2 cycles of ATG-010 drug therapy, the patient still had PD in October 2021. The patient received palliative radiotherapy for the right chest wall tumor starting in November 2021. After radiotherapy with dose of 36Gy/18F, the mass of chest wall significantly reduced in size. However, the patient discontinued treatment due to severe bone marrow suppression caused by radiotherapy and previous chemotherapy and died in May 2022.

### 2.1 Next-generation sequencing revealed tumor-specific mutations

To better understand the mutational landscape of the patient’s tumors, potential next‐generation sequencing (NGS) was performed on biopsy tumor tissue and blood samples. The most important findings were: TP53 gene copy number deletion (copy number: 0.8) and missense mutation of p.D281G exon 8 (abundance: 51.3%). Other abnormalities included a nonsense mutation of CD83 gene p.W49* exon 2 (c.147G>A, abundance: 43%), CDKN2A and CDKN2B gene copy number deletion (copy number: 0.4), a shear mutation in intron 8 of the FAS gene (c.676 + 1G>A, abundance: 34.3%), missense mutation of p.C479G exon 13 in LYN gene (c.1435T>G, abundance: 30.0%), and missense mutation of p.P84L exon 2 in PRDM1 gene (c.251C>T, abundance: 50.2%).

### 2.2 Single-cell transcriptomic analysis

#### 2.2.1 Identification of the five major cell types of DLBCL

**Figure 1:**
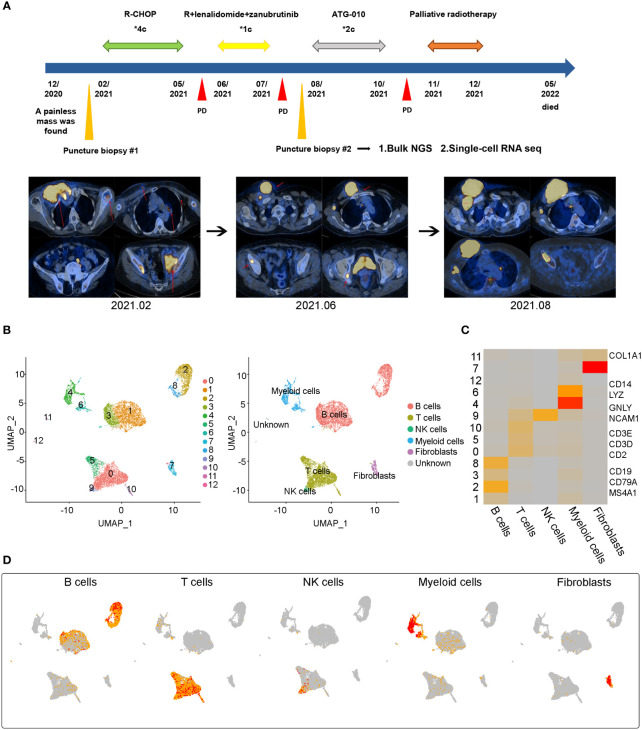
Case presentation and general overview of single cell transcriptome sequencing results in a refractory DLBCL sample **(A)** The treatment process of a 78-year-old woman and the representative CT images at the time of diagnosis (2021.02) and at two points of disease progression (2021.06 and 2021.08) **(B)** Uniform Manifold Approximation and Projection (UMAP) representation of twelve clusters and five identified cell types. **(C)** Heatmap of the relative expression level of marker genes across cells, sorted by cell type. Marker genes included CD19, CD79A and MS4A1 for B cells, CD2, CD3D and CD3E for T cells, GNLY and NCAM1 for NK cells, CD14 and LYZ for Myeloid cells, COL1A1 for fibroblasts. The expression was measured as the z-score normalized log2 (count+1). **(D)** Expression levels of typical marker genes across 9044 cells illustrated as UMAP plots.

After data quality control and filtering, a total of 9044 cells were analyzed. After dimensionality reduction and clustering, twelve major cell subpopulations were obtained using graph-based clustering (**Figure 1**). Five major cell types were identified using canonical marker genes: B cells (marker genes: CD19, MS4A1 and CD79A), T cells (marker genes: CD3D, CD3E, CD2), NK cells (marker genes: GNLY and NCAM1), myeloid cells (marker genes: LYZ and CD14), and fibroblasts (marker genes: COL1A1). Notably, B cells and T cells are the major cell subsets of DLBCL.

#### 2.2.2 Identification of malignant B cells

**Figure 2:**
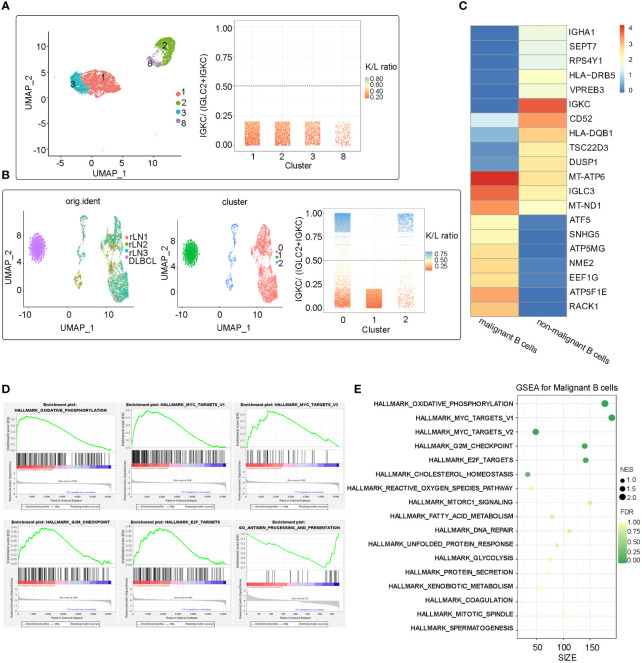
Malignant identification of B cells extracted from the refractory DLBCL patient’s tissue and integration analysis of benign B cells from external data **(A)** UMAP plot of 4361 B cells from the refractory DLBCL tissue, showing the formation of the 4 main clusters. The IGKC fraction, IGKC ÷ (IGKC + IGLC2), was calculated for each B cell. If the IGKC fraction was >0.5, we classified a B cell as κ+, and if this ratio was below 0.5, we classified the B cell as λ+. The percentage of B cells expressing either κ or λ was calculated per ranscriptionally distinct B-cell cluster. The non-malignant healthy B-cell cluster contained approximately 50% κ- and 50% λ-expressing B cells, whereas the malignant clusters contained B cells homogeneously expressing the λ or κ light chain. All the four B cell clusers (cluster 1, 2, 3 and 8) identified in this refractory DLBCL sample expressed the λ light chain homogeneously and were therefore identified as malignant B cells. **(B)** After integrating the scRNA-Seq data from the study of Roider et al., three clusters were identified after re-clustering. Cluster 0,1 from external single-cell data was identified as benign B cell population, Cluster 1 from our refractory DLBCL sample was identified as malignant. **(C)** Heat map showing the top 10 differential expressed genes in the malignant B cell subpopulation and non-malignant B cell subpopulations (Wilcoxon test). **(D)** Several significant pathways had higher or lower activities in malignant B cell subpopulation than non-malignant B cell subpopulations by Gene Set Enrichment Analysis (GSEA). **(E)** Bubble map of significant pathways enriched in the malignant B cells subpopulation by GSEA.

To investigate the transcriptomic heterogeneity of malignant B cells in DLBCL tissues, we re-clustered the B cells and identified four cell subpopulations (**Figure 2A**). To further distinguish malignant B cells from non-malignant B cells, we took advantage of the fact that the malignant B cell population expresses only one type of immunoglobulin light chain, i.e. κ or λ light chains (6). The IGKC fraction (IGKC/IGKC + IGLC2) method was used to distinguish malignant B cells (7). **Figure 2A** shows the IGKC fraction of the four B cell clusters 1, 2, 3, 8. It can be seen that the IGKC fraction of almost all B cells in these four clusters was lower than 0.25, indicating that they were B cells with uniform expression of λ+. Therefore, all four clusters of B cells were judged to be malignant B cells.

#### 2.2.3 Comparison of malignant and Normal B cells by expression profiling

No benign B cells were found in the DLBCL sample. We next compared malignant B cells from this patient with benign non-malignant B cells from a study by Roider et al. (three tonsil and reactive lymph node samples as control samples, named rLN1, rLN2 and rLN3, respectively (7). Differentially expressed genes (DEG) and gene set enrichment analysis (GSEA) were analyzed. Reunion and integration analyses were also performed. The Uniform Manifold Approximation and Projection (UMAP) showed that all B cells of the four samples were clustered into three clusters (0, 1, 2), and one malignant cluster (cluster 1) identified by the IGKC scoring method was well distinguished from the other two non-malignant clusters (cluster 0, 2) (**Figure 2B**).

DEG analysis revealed molecular disparity among malignant and non-malignant B cells. We constructed a heat map showing that the top 10 genes expressed in malignant B cells were related to ATP synthesis (ATP5F1E, ATP5MG, and ATP5MC2) and transcription regulation (ATF5, EEF1G, and ELOB), as well as LNCRNA (GAS5 and SNHG5) and ribosomal protein (RACK1) (**Figure 2C**). Notably, three of the ten genes that were significantly overexpressed were associated with ATP synthesis, which may be due to malignant B cells’ adjustment in response to metabolic stress in the DLBCL microenvironment. GSEA revealed that genes upregulated in malignant B cells were enriched in oxidative phosphorylation pathways and cancer-related pathways (MYC targets V1, MYC targets V2, G2/M checkpoint, E2F targets). In addition, HLA-DQB1 and HLA-DRB5, which are both MHC class II molecules, were significantly downregulated in malignant B cells suggesting immune escape in DLBCL. Furthermore, GSEA analysis showed that antigen processing and presentation enrichment were significantly downregulated in malignant B cells compared with normal B cells (**Figures 2D, E**). Taken together, these results indicate high levels of oxidative phosphorylation, activation of pro-tumor pathways such as MYC and E2F, and decreased immunogenicity caused by low expression of MHC molecules, which may be a key factor in initiating or accelerating oncogenic signaling in malignant B-cell carcinoma.

#### 2.2.4 Inter-transcriptomic heterogeneity of malignant B cells in DLBCL

**Figure 3:**
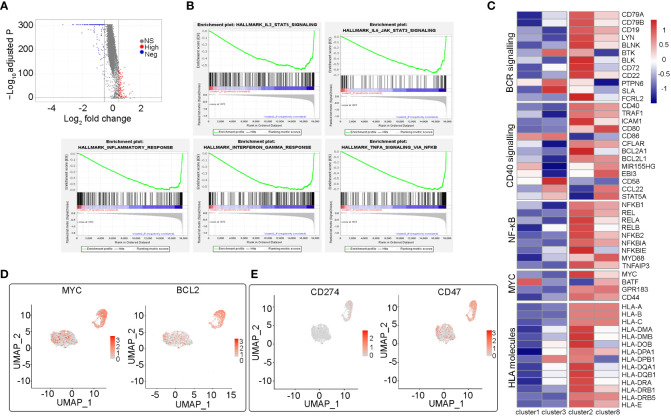
Heterogeneity analysis among two malignant B cell subpopulations (cluster2_8 vs cluster1_3) **(A)** The volcano map shows the differentially expressed genes between cluster2_8 and cluster1_3. **(B)** Several significant pathways with higher activities in cluster2_8 than cluster1_3 by GSEA. **(C)** Heatmap of the relative expression fold change (log2) of genes in essential pathogenic signaling pathways and HLA molecules in cluster2_8 and cluster1_3. **(D)** UMAP plots of selected genes (MYC, BCL2) expression level of different subsets of B cells. **(E)** UMAP plots of two immune checkpoint related genes (CD274, CD47) expression.

Using scRNA-seq, we identified two large malignant B cell subpopulations (including cluster1_3 and cluster2_8), showing high heterogeneity. The significantly differentially expressed genes in the cluster1_3 and cluster2_8 subpopulations were identified (avg_log2FC>=0.5 & p_val_adj <= 0.01) and the Volcano Plot was mapped (**Figure 3A**). Further GSEA analysis showed that hallmarks of TNFA signaling via NFKB, IL2/STAT5 signaling, IL6/JAK/STAT3 signaling, inflammatory response signaling, and interferon-gamma signaling were highly enriched in cluster2_8 compared with cluster1_3 (**Figure 3B**). In addition, we compared the expression levels of different pathogenic signaling pathways in B-cell lymphoma in two subpopulations and the results showed that expression of BCR signaling, CD40 signaling, and NFKB signaling was generally higher in cluster2_8 vs. cluster1_3. In addition, cluster1_3 showed low expression of HLA I and II molecules, which may be beneficial for evasion of immune surveillance (**Figure 3C**).

Double-expression lymphoma (DEL) refers to DLBCL with immunohistochemical evidence of the co-expression of MYC and BCL2. In addition to cell-of-origin (COO), microenvironment transcription markers, and some genetic drive markers, MYC and BCL2 double expression have also been used to classify DLBCL and predict prognosis (8, 9). The patient in this case was identified as having dual expression of MYC and BCL2 proteins by immunohistochemistry. Therefore, we analyzed MYC and BCL2 gene expression in B cell clusters and found that the expression of both two genes was significantly higher in cluster2_8 than in cluster1_3 (**Figure 3D**). Therefore, in the subsequent analysis, we named cluster2_8 as MYC+BCL2+ B cells, which were identified as MYC/BCL2 double expression subpopulations at the single-cell level, and cluster 1_3 as MYC-BCL2- B cells. In addition, the expression of CD274 and CD47, two immunosuppressive immune checkpoints, were higher in cluster2_8 than in cluster1_3 (**Figure 3E**). Overall, our results reveal a high degree of inter-tumor heterogeneity in DLBCL.

#### 2.2.5 Identification of immunosuppressive T-cell subsets

**Figure 4:**
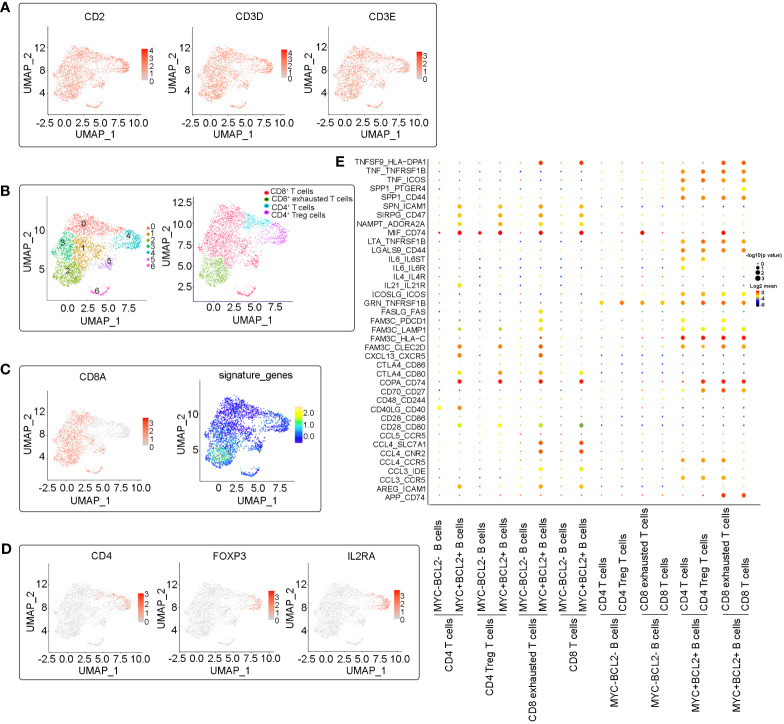
Subclustering analysis of T cells. **(A)** UMAP plots of expression of marker genes (CD2, CD3D and CD3E) in identified T cell clusters. **(B)** Identification and annotation of T cell subsets. Cells were colored according to their cluster or type. **(C)** UMAP plots plot showing the CD8 positive T cells clusters and exhausted-related signature genes (LAG3, PDCD1, TIGIT, HAVCR2, CTLA4, and TOX) expression in CD8 positive T cells clusters. **(D)** UMAP plots plot showing the expression of marker genes (CD4, IL2RA, FOXP3) in CD4 Treg cells. **(E)** Dot plot of predicted ligand-receptor interactions between different cell subsets in the microenvironments.

Tumor-infiltrating immune cells, especially T lymphocytes in the LME, are highly heterogeneous and play a crucial role in tumor immune evasion and immunotherapeutic efficacy. To study the intrinsic transcriptome characteristics of infiltrating T cells in DLBCL, particularly those with immunosuppressive properties, we re-clustered T cells and identified 10 CD4+ or CD8+ T cell subsets (**Figures 4A, B**). Increasing evidence has shown that a large number of regulatory T cells (Tregs) exist in tumor tissues, which are the main regulators of autoimmune tolerance. They inhibit anti-tumor immune responses by inhibiting cytokine production and inhibiting the proliferation of CD8+ T cells, which may lead to ineffective anti-tumor responses and the proliferation of cancer cells (10, 11). FOXP3+ Tregs have been reported to be associated with adverse outcomes in DLBCL (12, 13). In accordance with established markers (CD4+, IL2RA+, FOXP3+), the C2 cluster was identified as CD4+ Tregs (**Figures 4B, D**), which makes up a large percentage of CD4+ T cells. In addition, based on increased expression of exhausted markers (LAG3, PDCD1, TIGIT, HAVCR2, CTLA4, and TOX), we identified two typical exhausted CD8+ T cell clusters (C3 and C6, **Figures 4B, C**). Tregs cells and CD8+ exhausted T cells were significantly enriched in this relapsed/refractory patient, highlighting the microenvironment’s immunosuppressive nature. This finding suggests that this patient may benefit from immune checkpoint blockade therapies.

#### 2.2.6 Cellular communication in diffuse large B-cell lymphoma

In order to explore the internal crosstalk between malignant B cells and T cells, a ligand–receptor analysis was conducted. We analyzed the interactions between both MYC+BCL2+ B cells and MYC-BCL2- B cells with T cells, and found that MYC+BCL2+ B cells maintain the most frequent interactions with T cells (**Figure 4E**). TNF-TNFR2 and TNF-ICOS interactions were more enriched between MYC+BCL2+ B cells and T cells, suggesting that MYC+BCL2+ B cells may contribute to the maintenance of the tumor-friendly immune microenvironment. This is because TNF-TNFR2 and TNF-ICOS signals have been shown to recruit immunosuppressant immune cells (14–16). Similarly, MYC+BCL2+ B cells may inhibit T-cell proliferation via interactions with SPP1-CD44 (17). In addition, MYC+BCL2+ malignant B cells may induce an immunosuppressive microenvironment via SIRPG_CD47 (18), INAMPT_A2RA2A (19), and CTLA-4_CD80 (20) interactions. This suggests that an immunosuppressive microenvironment may be more likely in DLBCL overexpression of MYC and BCL2 than in DCBCL with negative or low expression of MYC and BCL2.

## 3 Discussion

In this study, we combined conventional bulk sequencing and single-cell sequencing with a high-resolution perspective to analyze DLBCL cells from a patient resistant to multi-course therapy. The results revealed that the causes of drug resistance were not only somatic mutations identified by bulk sequencing, but also heterogeneity among malignant cells and an immunosuppressive microenvironment.

TP53 is an important tumor suppressor gene (21). However, mutation of the TP53 gene leads to abnormal production of the p53 protein, which results in disordered proliferation of tumor cells and the emergence of drug resistance (21). TP53 mutations have been identified as a poor prognostic factor in DLBCL, and such patients do not respond well to standard first-line therapies (22, 23). TP53 mutation occurs in 20–25% of patients with DLBCL and is one of the most commonly mutated genes in this patient population (24). In this case, the patient was identified by NGS sequencing as having copy number deletion and a missense mutation (exon 8) in the TP53 gene, which may be a significant cause of drug resistance.

In addition to conventional NGS sequencing, we carried out single-cell sequencing, which can offer a high-resolution perspective to investigate intra-tumor heterogeneity and the tumor microenvironment. We identified malignant cell subpopulations with distinct transcriptional characteristics. First, we found that oxidative phosphorylation is significantly enriched in malignant B cells compared with normal B cells, suggesting that oxidative phosphorylation may be an essential factor in carcinogenesis. This is consistent with a previous study on DLBCL, which showed that DLBCL metabolism is heavily dependent on oxidative phosphorylation (OXPHOS) (25). This metabolic change can be beneficial in providing the energy needs of rapidly growing, high-grade lymphoma cells, and OXPHOS inhibition therapy appears to be effective in these tumor subtypes (26). In addition, hallmark pathways in malignant B cells were mainly enriched in the MYC TARGETS and E2F TARGETS, suggesting that MYC and E2F play essential roles in promoting the proliferation of DLBCL tumor cells.

Tumor heterogeneity is an important characteristic in tumor occurrence and development, especially in DLBCL, and an important factor in multi-drug resistance. We identified four malignant B cell clusters with different transcriptional information using scRNA-seq, suggesting a high degree of inter-tumor heterogeneity. Since clusters 1 and 3 and clusters 2 and 8 have transcriptional similarities, they were analyzed together. The continuous abnormal activation of the B cell receptor (BCR) signaling pathway is believed to be closely related to patient survival and the malignant proliferation of tumors. The inhibition of essential kinases in the BCR signaling pathway has become the main focus of drug development for B-cell lymphoma (27). Abnormal activation of the NF-κB pathway can upregulate the expression of multiple anti-apoptotic genes, including Bcl-2, TRAFs, and IAPs, which contribute to the continuous malignant proliferation of cells and the occurrence of cancer. Abnormal activation of the NF-κB signaling pathway has been previously demonstrated in DLBCL (27). Activation of CD40 signaling can enhance the survival of tumor B cells; therefore, targeting CD40 with a monoclonal antibody could inhibit this process (28). Our single-cell transcriptome data showed that the corresponding molecule expression of BCR signaling, CD40 signaling, and NF-κB signaling in cluster2_8 was generally higher than cluster1_3, suggesting that these pathways were more active in cluster2_8. Monoclonal antibodies or specific medications that target these pathways have emerged as potential anti-cancer therapies, such as BTK inhibitors and CD40 monoclonal antibodies. However, due to intra-tumoral heterogeneity, these drugs may not kill all tumor cells, leading to drug resistance and potentially relapse.

Loss of MHC expression on cancer cells represents one of the tumor immune evasion mechanisms and is usually associated with poor prognosis (29). Here, we showed that the MYC+BCL2+ B cells expressed higher levels of MHC class I and II genes than MYC-BCL2- B cells, suggesting weak immunogenicity of MYC-BCL2- B cells and thus inducing immune escape. Although MYC+BCL2+ malignant B cells show higher MHC expression, which increases immune system recognition and makes them more likely to induce “hot tumors” compared to MYC-BCL2- malignant B cells, some subsets of MYC+BCL2+ malignant B cells can actually create an immunosuppressive microenvironment. In our analysis, GSEA analysis showed that several inflammations and immune-related signaling pathways including IL2_STAT5_signaling, IL6_JAK_STAT3_signaling, inflammatory_response, interferon_gamma_response, and TNFA_signaling_via_NFKB were specifically enriched in cluster 2_8. Inflammatory responses play a pivotal role during tumor development, invasion, and metastasis (30). Consistent with our results, it was reported that tumor cells and stromal cells in DLBCL can promote inflammation and immunosuppression through IL6_JAK_STAT3 and NF-kB signaling and induce immune system evasion (31). We found that MYC+BCL2+ B cells can cause an inflammatory microenvironment with immunosuppressive characteristics through some immune inflammatory signaling pathways. This is consistent with the conclusion of recent studies, which showed that lymphoma cells with obvious proliferative characteristics have the potential to induce a ‘depleted’ microenvironment (32–34). The patient in this case was not only confirmed as a double-expressed patient by immunohistochemistry, but at the cellular level, we also identified a malignant B subgroup of “MYC+ BCL2+”, indicating high proliferation of B cells in the patient’s tumor.

Cell–cell interaction analysis also revealed that MYC+BCL2+ B cells seem to have more communications with T cells and may contribute to the maintenance of a tumor-friendly immune microenvironment through TNF-TNFR2, TNF-ICOS, SPP1-CD44, SIRPG_CD47, INAMPT_A2RA2A and CTLA-4_CD80 interaction (14–20). Exhausted CD8+ T cells and Tregs play an important role in the immunosuppressive microenvironment (10–13). Our analysis identified a high proportion of exhausted CD8+ T cells and FOXP3+ Tregs, which may indicate an immunosuppressive microenvironment induced by MYC+BCL2+ B cells interacting with T cells. This conclusion is consistent with two previous reports of immunosuppressive tumor microenvironments in DLBCL (35, 36). Immunotherapy, especially immune checkpoint inhibitors (ICIs), has made remarkable progress in the treatment of tumors. Depleted T cells express immunosuppressive receptors (such as LAG3, PDCD1, TIGIT, HAVCR2, CTLA4), and ICIs can block these signals, reverse depleted T cells, and restore the function of tumor-infiltrating T cells in the tumor microenvironment (37). Depending on the patient’s deep sequencing results, an immune checkpoint inhibitor, such as anti-PD-1 antibodies, alone or in combination with chemotherapy (if the blood hemogram permits) can then be tried.

In conclusion, by combining NGS and single-cell transcriptome sequencing technology, this study provides insight into somatic mutations, transcriptional features in malignant B cells, and the immune microenvironment landscape in a patient with muti-drug resistant DLBCL. The results revealed that several critical somatic mutations, highly heterogeneous tumor cells, and immunosuppressive tumor microenvironment jointly contribute to multi-drug resistance. This in-depth biological exploration can provide therapeutic targets and immunotherapy biomarkers for relapsed and refractory DLBCL patients.

## Data availability statement

The original contributions presented in the study are included in the article/supplementary materials. Further inquiries can be directed to the corresponding author.

## Ethics statement

The studies involving humans were approved by Medical Ethics Committee of Tongji Medical College, Huazhong University of Science and Technology. The studies were conducted in accordance with the local legislation and institutional requirements. The participants provided their written informed consent to participate in this study. Written informed consent was obtained from the individual(s) for the publication of any potentially identifiable images or data included in this article.

## Author contributions

KZ: Formal analysis, Methodology, Resources, Validation, Writing – original draft, Writing – review & editing. QL: Formal analysis, Methodology, Software, Validation, Writing – original draft, Writing – review & editing. PL: Methodology, Software, Validation, Writing – review & editing. TL: Supervision, Writing – review & editing. XL: Supervision, Writing – review & editing. FZ: Supervision, Writing – review & editing. LZ: Conceptualization, Project administration, Validation, Writing – review & editing.
